# Association of Mucosal Organisms with Patterns of Inflammation in Chronic Rhinosinusitis

**DOI:** 10.1371/journal.pone.0136068

**Published:** 2015-08-14

**Authors:** Thanit Chalermwatanachai, Nan Zhang, Gabriele Holtappels, Claus Bachert

**Affiliations:** 1 The Upper Airways Research Laboratory, Department of Oto-Rhino-Laryngology, Ghent University Hospital, Ghent, Belgium; 2 Department of Otolaryngology, Phramongkutklao Hospital and College of Medicine, Bangkok, Thailand; 3 Division of ENT Diseases, Clintec, Karolinska Institute, Stockholm, Sweden; Universidade Federal do Rio de Janeiro, BRAZIL

## Abstract

**Background:**

Chronic rhinosinusitis is a multifactorial process disease in which bacterial infection or colonization may play an important role in the initiation or persistence of inflammatory response. The association between mucosal bacteria presence and inflammatory patterns has only been partially explored.

**Objective:**

To demonstrate specific mucosal microorganisms possible association with inflammatory patterns.

**Methods:**

We collected nasal polyps or sinus tissues from a clinical selection of six patient groups with defined sinus disease using tissue biomarkers. In the tissues, we detected bacteria using peptide nucleic acid fluorescence in situ hybridization (PNA-FISH).

**Results:**

After reviewing a total of 115 samples (15–20 samples per group), the mucosal presence of *Staphylococcus aureus* was correlated with IL-5 and SE-IgE positive chronic rhinosinusitis with nasal polyps and nasal polyps from cystic fibrosis patients. Chronic rhinosinusitis without nasal polyps with TNFα >20 pg/ml was associated with the mucosal presence of *Pseudomonas aeruginosa*.

**Conclusion:**

This study identifies the relationship between intramucosal microbes and inflammatory patterns, suggesting that bacteria may affect the type of inflammation in chronic rhinosinusitis. Additional investigation is needed to further identify the nature of the relationship.

## Introduction

Rhinosinusitis is a broad general term used to describe various inflammatory diseases of the nasal and paranasal cavities. The European Position Paper on Rhinosinusitis and Nasal Polyps (EPOS) concurs that rhinosinusitis is characterized by two or more symptoms: either nasal blockage/obstruction, or nasal discharge; facial pain/pressure, reduction or loss of smell, or both [1]. The chronic status of the disease is defined when symptoms persist at least 12 weeks without complete amelioration.

The role of infectious agents in patients with acute rhinosinusitis (ARS) is well established but the role in chronic rhinosinusitis (CRS) remains unclear. CRS affects 10.9% of the European population, which increases direct medical costs and affects overall general health outcomes [2].

CRS can be classified into two clinical subgroups (phenotypes) based on the absence and presence of nasal polyps (CRSsNPs and CRSwNPs) [1]. These two subgroups may be further divided into several disease endotypes based on the cytokine composition predominant within the tissue [3]. The phenotype is useful in treatment planning while the endotype provides insight in pathophysiological mechanisms of CRS. Therefore, investigations of the pathogenesis and the factors amplifying mucosal inflammation are therefore, of crucial importance for the development of new diagnostic and therapeutic tools.

In our previous findings, using the peptide nucleic acid-fluorescence in situ hybridization (PNA-FISH), we reported a higher quantity of *Staphylococcus aureus* (*S*. *aureus*) in nasal polyps (NPs) tissues from patients with aspirin exacerbated respiratory disease [4]. We also found an association between gram-positive bacterial colonization and interleukin (IL)-5-positive NPs, whereas gram-negative bacteria were associated with IL-5-negative NPs [5]. Thus, specific inflammatory endotypes are likely to be associated with the intramucosal presence of specific bacteria but it remains unclear which organisms are related to the different endotypes.

Microorganisms have a possible influential role at least in selected subsets or endotypes of CRS; mucosal bacteria might have a specifically direct and strong impact on the mucosal immune system. To validate this hypothesis, we attempted to illustrate the association of specific microorganisms and specific inflammatory patterns. The main emphasis of the current study was on intra-mucosal microorganisms such as *S*. *aureus*, *Pseudomonas aeruginosa* (*P*. *aeruginosa*) and *Escherichia coli* (*E*. *coli*), and fungi all detectable by the PNA-FISH.

## Materials and Methods

### Nasal tissue

Nasal tissue samples (n = 115) with defined disease and pathology were selected from a tissue collection at the Department of Otorhinolaryngology, Ghent University Hospital. The study for collecting human tissue samples was approved by the ethics committee of the University of Ghent, Belgium and appointed the number B67020111209. All patients gave written informed consent before being operated on. These samples were obtained from patients during endonasal sinus surgery. The inferior turbinates from patients undergoing septoplasty without sinus disease were used as control. The diagnosis of CRS in each patient was in compliance with the EPOS guidelines. Diagnosis of cystic fibrosis (CF) and asthma was subsequently confirmed by a pediatrician/pulmonologist. The atopic status was evaluated using the Phadiotop test (Phadia; Uppsala, Sweden). Cytokine profiles were performed on all disease tissue samples. CRS tissue samples were classified according to their cytokine composition in five groups: sinus tissues from CRSsNPs patients with tumor necrosis factor alpha (TNFα) mucosal concentrations <20pg/ml or >20pg/ml, CRSwNPs tissues with IL-5 and SE-IgE expression, nasal polyp tissues without IL-5 and SE-IgE expression, and nasal polyps from patients with CF.

### Measurement of Cytokines in Tissue

Snap-frozen tissue specimens were weighed and suspended in a ratio 0.1 g of tissue per 1 mL of 0.9% NaCl solution with a complete protease inhibitor cocktail (Roche; Mannheim, Germany). To prepare soluble protein fractions, the frozen tissues were pulverized using mechanical Tissue Lyser LT (Qiagen Hilden, Germany) at 50 oscillations per second for two minutes in prechilled Eppendorf tubes. Homogenized tissues were centrifuged at 15,000 rpm for five minutes at 4°C, and the supernatants were collected. Total IgE, eosinophil cationic protein (ECP) and specific IgE to staphylococcal enterotoxins (SE-IgE) were measured using the UniCAP system (Phadia; Uppsala, Sweden). Assay of the TNFα, interleukin (IL)-5, and IL-17 were performed using Luminex (R&D system; Minneapolis, MN, USA). Interferon gamma (IFNγ) was determined using ELISA (R&D system; Minneapolis, MN, USA). IL-22 was assayed using Duoset-Elisa from R&D system.

### Identification of Microorganisms by Peptide Nucleic Acid Fluorescence in situ Hybridization

Nasal mucosal tissues were prepared using frozen section procedure and cut to 5μm, fixed with 70% ethanol for ten minutes and air-dried. Subsequently, the dried sections were hybridized at 55°C for 90 minutes in a humidified chamber with 100–500 nM of fluorescein-labeled PNA probe (Uniprobe, which hybridizes with a wide range of gram positive and gram negative bacteria; *S*. *aureus*; *P*. *aeruginosa* and *E*. *coli*; and panFungus PNA-FISH kit; AdvanDx; Woburn, MA, USA). After hybridization, the coverslips were removed by submerging each slide in the stringent wash buffer provided in the kit and washed for 30 minutes in a shaking water bath at 55°C. Each slide was subsequently mounted in Vectashield (Vector; Burlingame, CA, USA) containing 4,6-diamidino-2-phenylindole dihydrochloride (Roche Molecular Biochemicals; Brussels, Belgium) to counterstain the nuclei. A negative control (without PNA probe) was run in parallel to each sample. Microscopic examination was conducted with a Zeiss Axioplan epifluorescence microscope (Carl Zeiss; Gottingen, Germany) equipped with a CCD camera (IMACCCD S30; SONY, Germany) using a fluorescein isothiocyanate specific filter. Images were captured using the Isis imaging and software system (MetaSystems; Sandhausen, Germany). The samples were assessed using a score of bacterial appearance in the mucosa from 0 to 3: 0, negative; 1, extraepithelial presence; 2, intraepithelial presence and 3, subepithelial presence. Ten high-power fields were counted and added resulting in a total mucosal score between 0 and 30. Furthermore, we interpreted mucosal invasion as; negative (all 10 fields scored 0), noninvasive (at least 1 field scored 1), or intramucosal invasive (at least 1 field scored 2 or 3) based on the maximum score. Two independent observers, not privileged to the diagnosis and clinical data, evaluated the slides.

### Statistical Analysis

Statistical analysis was performed with GraphPad Prism version 6.00 for Mac OS X (GraphPad Software; La Jolla CA, USA, www.graphpad.com). The categorical data (sex, positive atopy, asthma, and probe) were expressed as frequencies or percentages and were analyzed using the Chi-square test. The interval data (age, level of cytokines, and total mucosal score) were tested for distribution using the Shapiro-Wilk normality test revealing that the data were distributed non-normally. The Kruskal-Wallis test was used to assess the significance of intergroup correlation, and corrections of significance for between-group comparisons were calculated using Dunn's test. The Spearman’s rank correlation was employed to assess the statistical correlation between cytokine and mucosal score. The statistical significance level was determined as *p* <0.05.

## Results

### Characteristics in the Nasal Tissue Samples

Table 1 illustrates the number of samples and clinical characteristics of the patients. More than half (62.5%) of the patients with IL-5 and SE-IgE positive NPs had co-morbid asthma. CF patients were the youngest as expected. No significant differences were found with regard to sex and atopy in intergroup comparisons of patients.

**Table 1:** Sample characteristics.

	Control	CRS TNFα<20	CRS TNFα>20	NPs IL5&SE-IgE Neg	NPs IL5&SE-IgE Pos	NPs Cystic Fibrosis	Statistic
**No. of sample**	20	20	20	20	20	15	
**Mean age (yr)**	32.75	44.45	39.35	41.05	51.2	13.25	*p*<0.0001
**Female/male**	5/15	11/9	11/9	10/10	8/12	6/9	NS *p* = 0.3691
**Phadiotop positive**	5.88%	20%	31.25%	29.41%	52.94%	n/a	NS *p* = 0.6776
**Asthma**	0%	20%	20%	35.29%	62.50%	n/a	*p* = 0.0330

### Cytokine Patterns in the Nasal Tissue Samples

**Fig 1:**
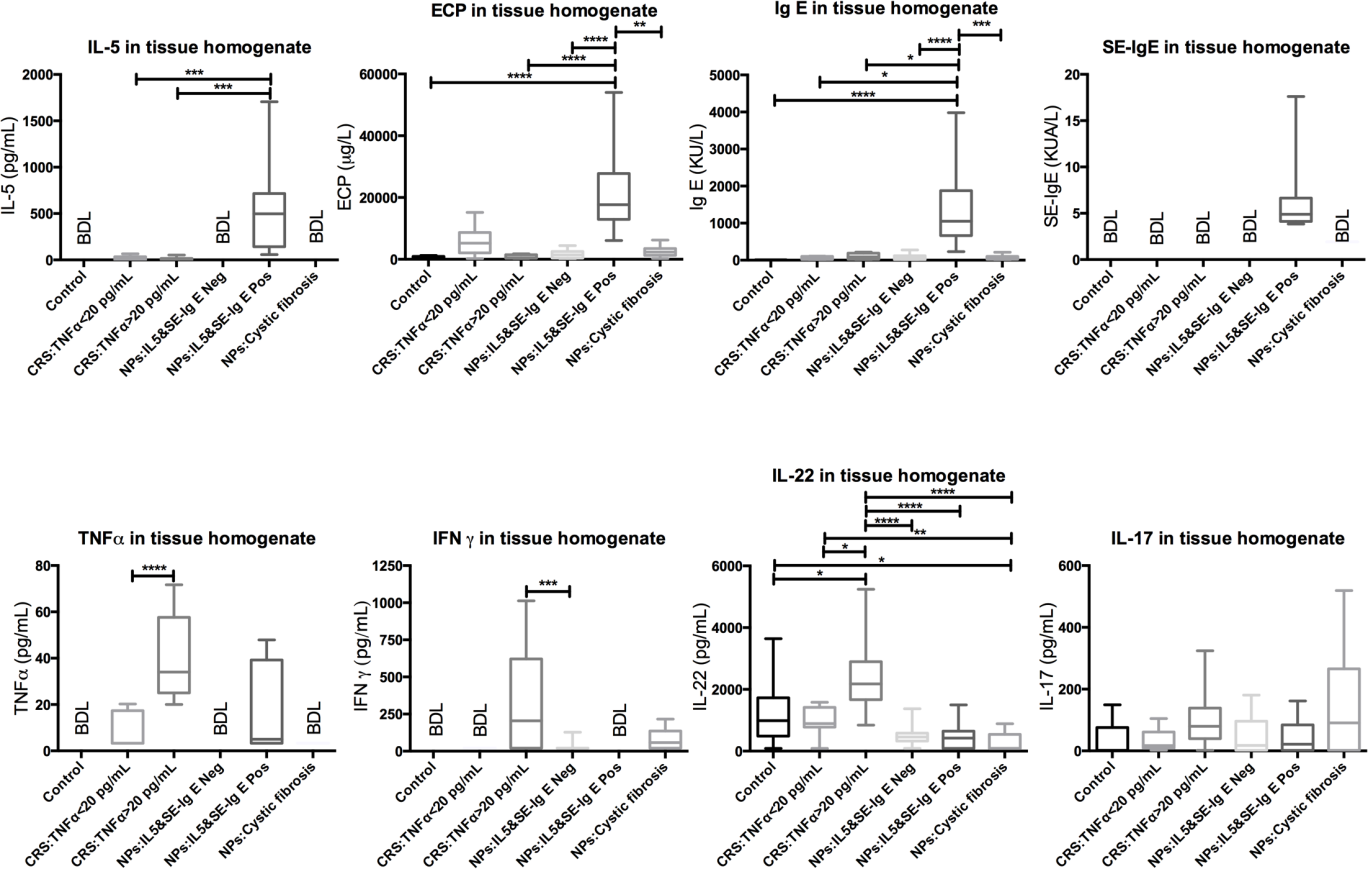
Cytokine expression in sinus tissues. The graphs represent median, upper and lower quartiles. High IgE, IL-5 and ECP concentrations characterize the inflammation in IL5&SE-IgE positive NPs. In CRS with TNFα >20 pg/mL, the expression of IFNγ and IL-22 are elevated. Statistical analysis was performed using Kruskal-Wallis and Dunn’s multiple comparisons test. An asterisk (*) refers to *p* values ≤0.05. The *p* values between 0.001 and 0.01 are displayed with two asterisks (**), *p* values between 0.0001 and 0.001 are shown with three asterisks (***), and *p* values less than 0.0001 are labeled with four (****) asterisks.

Typical patterns of cytokines for CRSsNPs are T helper 1(Th1)-driven, whereas CRS with eosinophilic NPs is T helper 2(Th2)-biased and CRS with neutrophilic NPs expresses increased amounts of IFNγ and/or IL-17 or none of those. NPs from CF patients demonstrate high concentrations of IL-17 and IL-8 [6]. In the current study, concentrations of ECP and IgE were significantly higher in NPs with IL-5 expression compared with all other groups. Additionally, an increase in SE-IgE concentration was observed. The number of SE-IgE positive subjects was significantly higher in this group compared with the controls and the other groups. TNFα was detected in sinus tissues from CRS patients and IL-5 positive NPs. IFNγ was noted in the sinus tissue homogenates with a concentration of TNFα >20 pg/ml, IL-5 negative NPs, and CF-NPs. The expression of IL-22 showed a significant increase in the TNFα >20 pg/ml sinus tissue homogenates compared with all other groups. A remarkably decreased level of IL-22 was observed in NP from CF patients compared with the control and CRSsNP patients. Although IL-17 expression was higher in NP from CF patient, it did not reach the level of statistical significance (*p* = 0.0545)(Fig 1).

### PNA-FISH Probe Results

**Fig 2:**
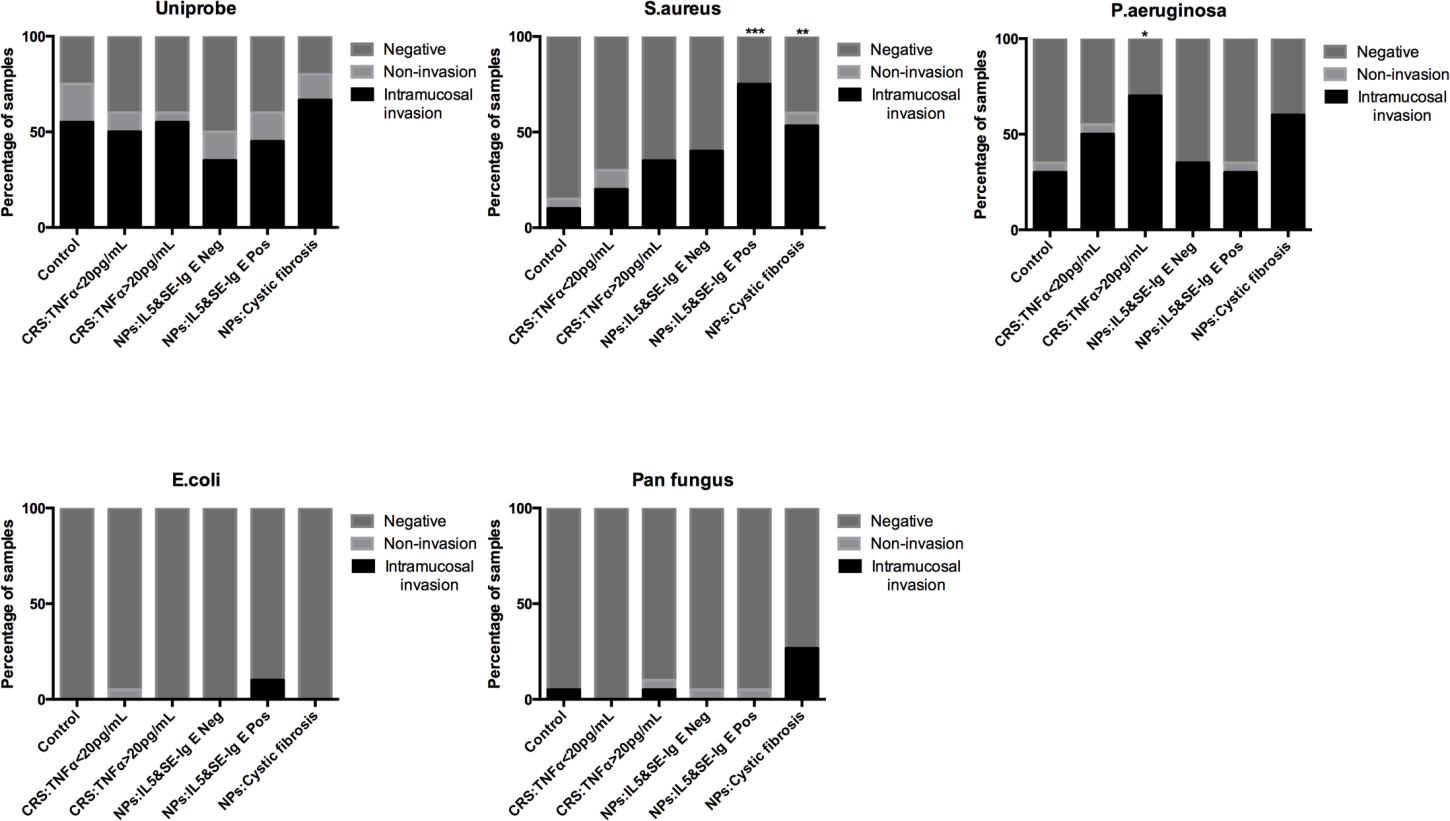
PNA-FISH. The graphs illustrate percentages of samples of bacterial presence for each of the probes in the different patient groups. The highest percentage of positive samples for *S*. *aureus* was identified among the IL5&SE-IgE positive NPs and patients with NPs and CF, whereas *P*. *aeruginosa* was discovered in high numbers in CRS samples with TNFα>20pg/mL. Differences from the control group were calculated using the Chi-square test. The * p values ≤0.05, ** *p* values between 0.001 and 0.01,*** *p* values between 0.0001 and 0.001 were recorded

*S*. *aureus* was detected in a number of samples from the IL-5 and SE-IgE positive NPs (75%) and CF-NPs groups (60%) compared with the control group (15%, *p* = 0.0001 and *p* = 0.0055 respectively). The presence of *P*. *aeruginosa* in samples of sinus tissue with TNFα concentrations >20 pg/ml (70%) was significantly higher than in the control group (35%, *p* = 0.0267). The PNA-FISH probe for *E*. *coli* and panfungus revealed no statistically significant difference among all groups. Furthermore, the Uniprobe discovering all bacteria did not show any difference in numbers, indicating that all samples had about the same total bacterial load. Representative PNA probe findings are shown in Fig 2.

**Fig 3:**
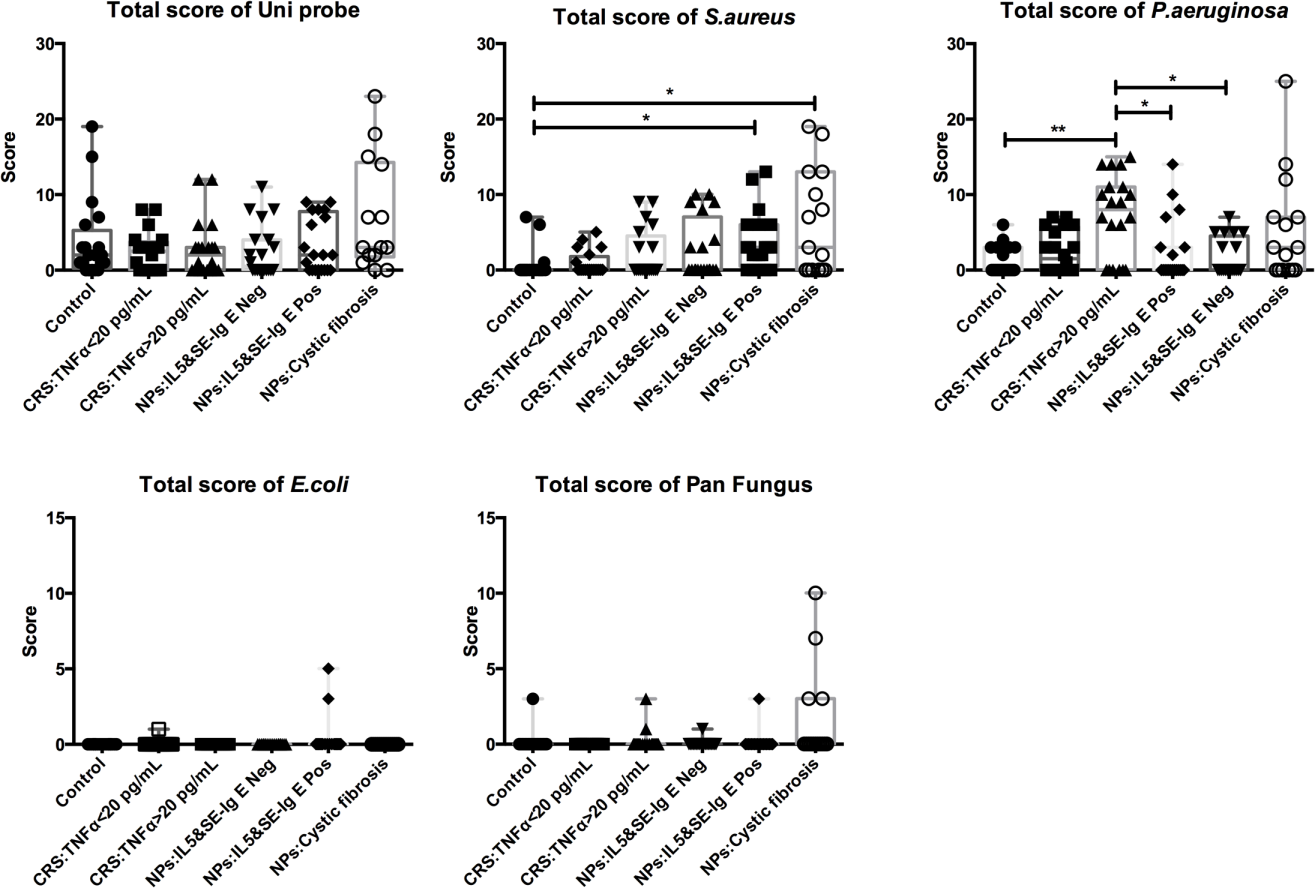
Total mucosal scores for the presence of bacteria in sample tissues, detected by PNA-FISH. Each point represents the score from the individual sample. The data for each group are expressed as median and interquartile range. The total score of *S*. *aureus* was specifically high in IL5&SE-IgE positive NPs and CF-NPs compared with the controls. CRS with TNFα >20 pg/mL showed the highest score for *P*. *aeruginosa* compared with the controls and NPs, but not with CF-NP. The significance between groups was tested using Kruskal-Wallis test. Dunn’s test was employed to correct the significance for between-group comparisons. The * *p* values ≤ 0.05, ** *p* values between 0.001 and 0.01 were recorded.

The total mucosal scores (median [interquartile range]) for *S*. *aureus* in the IL-5 and SE-IgE positive NPs (3 [0–13]) and among CF-NPs (3 [0–19]) were significantly different from the control group (0[0–7]). Asthma had no significant impact on the scores in the IL-5 and SE-IgE positive NPs. Invasion of the mucosa by *P*. *aeruginosa* was significantly more frequent in the TNFα >20 pg/ml sinus tissues (median 8 [0–15]) compared with control tissues (median 0 [0–6])(Fig 3).

### Correlations between Cytokine Levels and Total Mucosal Score

**Fig 4:**
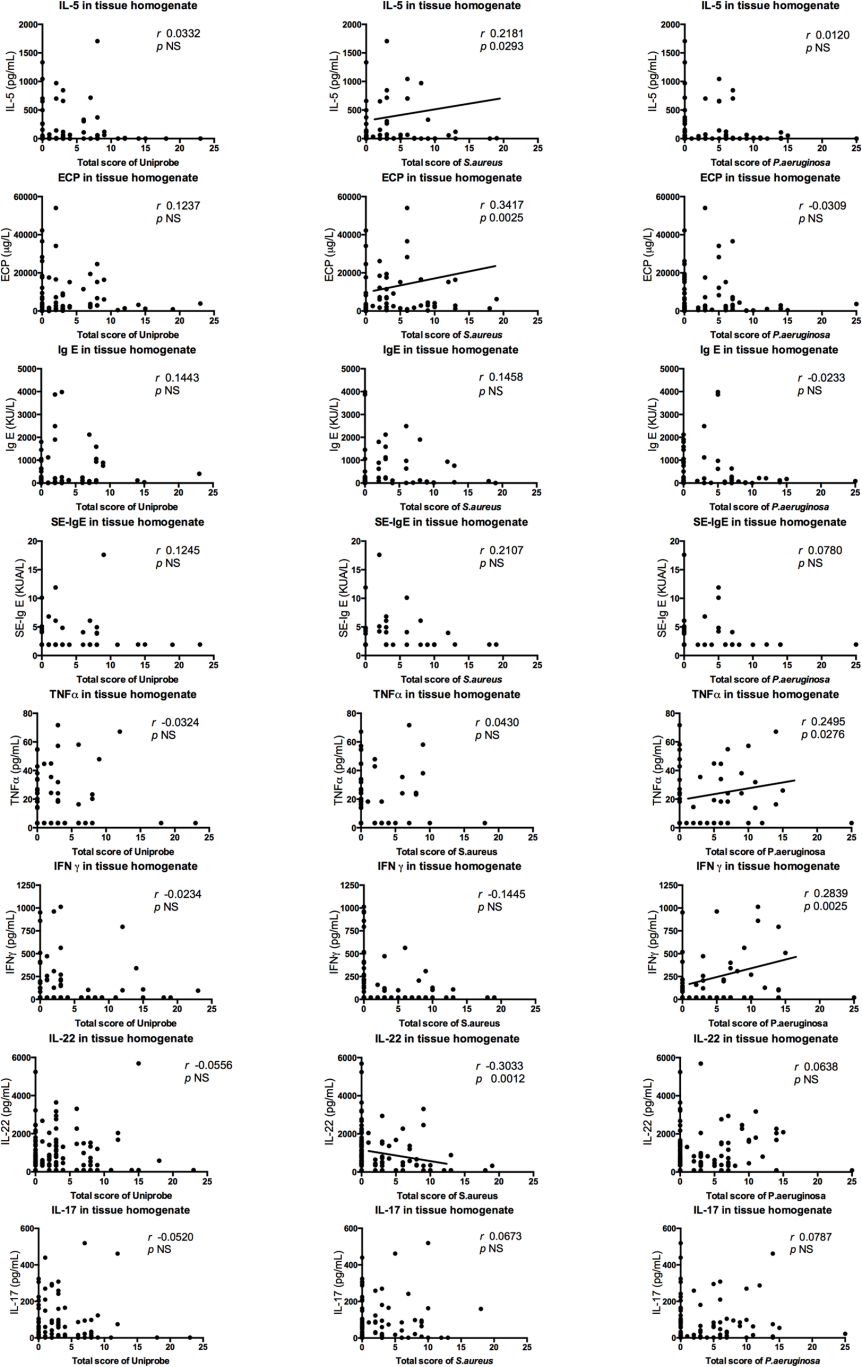
The correlations between cytokines and total mucosal scores of each PNA-probe. The correlations were calculated using Spearman’s rank test. Correlation coefficient (*r*) and *p* value were given for each correlation. NS stands for Not Significant (*p* >0.05).

Correlations of cytokines and scores of bacterial appearance in the mucosa reveal that IL-5 and ECP were positively correlated with the *S*. *aureus* score (*r* = 0.2181, *p* = 0.0293; and *r* = 0.3417, *p =* 0.0025, respectively). In contrast, IL-22 was negatively correlated with the *S*. *aureus* score (*r* = -0.3033, *p* = 0.0012). The *P*. *aeruginosa* score positively correlated with TNFα (*r* = 0.2495, *p* = 0.0276) and IFNγ (*r* = 0.2839, *p* = 0.0025). However, no cytokine detectable in tissue correlated with the Uniprobe score (Fig 4).

## Discussion

Using PNA-FISH detection we demonstrated that the presence of *S*. *aureus* was associated with IL-5 and SE-Ig E positive CRSwNPs and NPs from patients with CF, whereas *P*. *aeruginosa* was specifically found in the nasal tissues of CRSsNPs with TNFα concentrations above 20 pg/ml.

*S*. *aureus*, a gram-positive cocci bacterium, expresses a range of virulence factors that challenge the immune system [7]. Our results were consistent with earlier findings that *S*. *aureus* could drive Th2 type inflammation [3, 5, 8]. The association of *S*. *aureus* superantigens and CRSwNPs was recently confirmed in a meta-analysis [9]. Furthermore, our findings confirm that *S*. *aureus* colonized the airways of young patients with CF [10]. However, whereas mucosal *S*. *aureus* enterotoxin-specific IgE could be found in adult patients with CRSwNPs, those antibodies could not be detected in patients with CF. The reasons for this observation may lie in the ability of *S*. *aureus* to produce enterotoxins or in the immunological environment. For CRSwNPs, the presence of IL-5 and SE-Ig E but not *S*. *aureus* in tissue was associated with comorbid asthma [4, 11]. Moreover, the level of IL-5 and SE-Ig E was demonstrated to be significantly increased in recurrent versus nonrecurrent CRSwNPs [12].

*P*. *aeruginosa* is a gram-negative, motile rod bacterium. This study revealed the clinical meaningful presence of *P*. *aeruginosa* in sinus tissues with TNFα concentrations higher than 20pg/ml, coinciding with elevated levels of IFNγ and IL-22. These findings confirm a Th1 milieu in CRSsNPs and suggest that *P*. *aeruginosa* may drive Th1 type inflammation and a specific antibacterial response [13]. *P*. *aeruginosa* does not only stimulate TNFα production in epithelial cells [14], but also induces dendritic cell maturation to the Th1-polaizing phenotype [15]. Moreover, *P*. *aeruginosa* activates dendritic cells, Th1 cells, and group 3 innate lymphoid cells (ILC) in the mucosa to produce IL-22 [16, 17]. IL-22, a member of the IL-10 family [18] is essential for mucosal immunity. It boosts the innate immunity [17] and induces epithelial cells to produce antimicrobial proteins [19]. In murine models, IL-22 was demonstrated to play a vital role in the fight against gram-negative bacteria in the respiratory system [20, 21]. The decreased expression of IL-22 in CF-NPs may predispose these patients to gram-negative infection in adults. This study illustrated that intramucosal *P*. *aeruginosa* colonization relates to TNFα and IFNγ levels in the tissue. TNFα has been defined as a key regulatory cytokine of inflammatory responses and plays an important role of normal host defense against microorganisms [22]. Our data suggest that either TNFα or IFNγ may serve as markers for *P*. *aeruginosa* infections in CRSsNPs.

Uniprobe detects a broad range of bacteria. The fact that we found equivalent amounts of bacteria in control and diseased tissues was supported by recent findings on the sinus microbiome, showing similar results [23]. Whereas the number of germs per surface area may be similar, the richness and evenness of these germs differentiate healthy from diseased conditions. The intramucosal presence of bacteria has been not only demonstrated before in disease, but also in healthy conditions [4, 24].

The prevalence of fungi in CRS has long been considered. In our experiment, the pan-fungal PNA-FISH probe only detected a small proportion of fungi without differences between groups by hybridizing with 28S rDNA genes [25]. Although studies have reported a predominance of fungi in CRS patients [26, 27], the prevalence of fungal DNA in CRS patients was shown to be lower than 10% [28]. Although it should be taken into account that we have slightly underestimated the number of fungi present due to the failure of PNA-FISH probes to penetrate the rigid fungal cell walls [29], this study disproves the hypothesis that fungi are principal offenders in the development of CRS.

Future directions for research should involve 16S rRNA gene pyrosequencing and quantitative real-time PCR approaches and study the immune proteomes of the germs and the pathomechanisms involved in the crosstalk with the host. Exploration of the relationships between microbes and their impact on sino-nasal immune responses may lead to a greater understanding of the pathogenesis of CRS, and thus, result in new strategies for its treatment.

## Conclusion

For the first time, this study identified a relationship between intramucosal microorganisms and inflammatory patterns in subgroups of patients with CRS. Variations of intramucosal bacteria have been identified between the different CRS endotypes. *S*. *aureus* is associated with Th2-bias CRSwNPs and NPs from patients with CF while *P*. *aeruginosa* is related to high inflammatory CRSsNPs. We propose TNFα and/or IFNγ as a biomarker for CRSsNPs. The interaction of microbes with the mucosal immune responses should be further evaluated.

## Supporting Information

S1 File The primary data of this study. (XLS)
